# Editorial: Food-derived phytochemicals as regulators of gut microbiota

**DOI:** 10.3389/fnut.2025.1681732

**Published:** 2025-08-29

**Authors:** Samanta Thomas-Valdés, Gonzalo Jorquera

**Affiliations:** ^1^Centro de Micro-Bioinnovación, Escuela de Nutrición y Dietética, Universidad de Valparaiso Facultad de Farmacia, Valparaíso, Chile; ^2^Instituto de Nutrición y Tecnología de los Alimentos (INTA), Universidad de Chile, Santiago, Chile; ^3^Instituto de Fisiología, Facultad de Ciencias, Universidad de Valparaíso, Valparaíso, Chile

**Keywords:** gut microbiota, phytochemical, gut-liver axis, gut-bone axis, gut metabolites, short-chain fatty acids, fecal microbiota transplant (FMT), aging

## Introduction

Plants supply a dazzling repertoire of bioactive compounds—polyphenols, carotenoids, terpenoids, alkaloids, organosulfur compounds, and many others—now exceeding 5,000 cataloged structures (1, 2). Cohort and intervention data consistently link phytochemical-rich diets to lower risk of obesity, insulin resistance, type 2 diabetes, cardiometabolic disease, several types of cancers, and neurodegeneration (3). While their redox and anti-inflammatory chemistry are well known, mounting evidence shows that many benefits are microbiota-mediated (4, 5). Therefore, this Research Topic explores how food-derived phytochemicals remodel the gut ecosystem and, through it, human physiology.

## Diet–microbiota crosstalk

Phytochemicals reach to the colon largely intact where they are metabolized by intestinal microbiota reshaping community structure, enriching health-associated taxa while suppressing pathobionts (6, 7). Such restructuring is not a side effect but a driver of benefit: the new community generates metabolites—short-chain fatty acids (SCFAs), secondary bile acids, indole derivatives—that reinforce tight junction proteins, lower colonic pH, and signal via receptors such as G-protein–coupled FFARs, FXR/TGR5, or the aryl-hydrocarbon receptor (8). These host–microbe co-metabolites dampen inflammation, improve insulin sensitivity, and fine-tune lipid metabolism, creating a virtuous cycle in which phytochemicals act as both substrates and regulators of gut bacteria (6, 9).

## Lessons from microbiota transplantation

Lamas-Paz et al. extend this concept beyond diet. In their alcohol-challenge model, middle-aged male mice showed gut leakiness, dysbiosis, and severe liver injury—phenotypes largely reversed after fecal microbiota transplantation (FMT) from age-matched females (Lamas-Paz et al.). Tight-junction proteins (ZO-1, occludin), mucus (MUC2), and toll-like-receptor signaling normalized; hepatic steatosis, inflammation, and senescence markers fell sharply. Though no plant compounds were used, the study underscores a unifying principle: engineering microbial communities—whether by feeding targeted phytochemicals or seeding a health-associated consortium—can stabilize the gut barrier and blunt systemic injury (Lamas-Paz et al.). FMT thus marks the intensive end of a continuum on which polyphenol-rich foods operate more gently, hinting that future precision-nutrition strategies may mix both levers, calibrated to sex, age, and exposure history.

## Phytochemicals in action

Direct evidence comes from Wilson et al., who administered polyphenol-dense *Aronia melanocarpa* juice to humanized mice bearing microbiota from donors with low- (LO) or high-inflammation (HI) phenotypes. Aronia juice shielded mice against high-fat-diet dysmetabolism, expanded an *Eggerthellaceae* genus ~7-fold, and boosted phosphatidylcholines linked to barrier integrity. Crucially, LO donor communities preserved β-diversity and resisted global metabolomic shifts far better than HI communities, illustrating that phytochemical efficacy is context-dependent—the food matrix and the starting microbiome jointly dictate benefit.

In the same way, saponins from *American ginseng*, recognized by their potent antioxidant properties, reshaped positively the gut microbiota from aged mice. A single intervention and combined intervention of Rb1 and Re saponins enhanced the α-diversity of gut microbiota, especially when combined Rb1 + Re, recovering include to the level of young mice. Such saponins can promote the abundance of probiotics, including *Lactobacillus, Lactobacillaceae*, and *Bifidobacterium*, and inhibit harmful bacteria such as *Enterobacteriaceae* (Shi et al.).

Polysaccharides obtained from plants are emerging as detoxifying agents explained in part by their impact in the gut microbiota modulation. In a Cadmium (Cd)-induced liver injury model, *Polygonatum sibiricum* polysaccharides (PSP) supplementation reduced serum alanine aminotransferase (ALT) and aspartate aminotransferase (AST) levels, improved hepatic steatosis, increased intestinal villi height, enhanced intestinal barrier function, promoted the growth of beneficial bacteria (*Lactobacillus*), besides modulate the production of SCFAs. Such effects alleviated hepatic dysfunction and metabolic disorders. Di et al. demonstrated the PSP potential as a functional dietary intervention for alleviating hepatotoxicity throughout gut-liver axis.

Extending this phytochemical-first lens to the gut–bone axis, Wei et al. argue that many osteoprotective effects ascribed to “nutraceuticals” are in fact co-productions of diet and microbes. SCFAs and microbe-enabled biotransformation of dietary polyphenols—e.g., isoflavones → equol, ellagitannins → urolithins, and lignans → enterolignans—engage estrogen receptors, promote osteoblastogenesis, and restrain osteoclast activity, thereby reinforcing bone homeostasis. Converging evidence also shows that plant-derived prebiotics, such as grape-seed anthocyanins and konjac oligosaccharides, can enrich *Bifidobacterium*, restore barrier function, and recalibrate immune tone—an ecological route to bone protection that complements polyphenol intake. From this perspective, next-generation probiotics and bacterial extracellular vesicles (BEVs) are best viewed as a delivery layer atop diet—stabilizing the production and targeting of beneficial metabolites along the gut–bone axis—rather than as stand-alone fixes.

## Future directions

Several gaps remain—and they mirror the themes of this Research Topic. First, we need human dose–response trials that report not only clinical endpoints but also mechanistic readouts (SCFAs, secondary bile acids, indole derivatives), barrier markers (ZO-1, occludin, MUC2), and axis-specific outcomes (gut–liver, gut–bone). Second, because efficacy is context-dependent, trials should stratify or adjust by baseline microbiome/inflammation phenotypes (e.g., in LO/HI donors), sex and age, and routinely record concomitant modifiers (antibiotics, proton pump inhibitors, metformin, ultra-processed diet). Third, we need to map keystone taxa and enzymes that unlock specific phytochemicals (e.g., equol, urolithins, enterolignans) and determine how food matrices (fiber, saponins, polysaccharides) shape bioavailability and microbial metabolism. Fourth, combined strategies deserve testing: dietary phytochemicals as the foundational lever, complemented—when appropriate—by microbiota-targeted tools (probiotics, symbiotics, BEVs, or even FMT) to stabilize metabolite production and barrier integrity. Finally, to improve comparability and translation, studies should standardize phytochemical characterization and dosing, include multi-omics (metagenomics, metabolomics, host transcriptomics), ensure safety monitoring, and adopt open protocols and data. Taken all together, these steps will move the field from associative signals to actionable, precision nutrition, turning the microbiome–phytochemical dialogue into reproducible health gains.
